# Efficacy of Different Drenching Regimens of Gluconeogenic Precursors during Transition Period on Body Condition Score, Production, Reproductive Performance, Subclinical Ketosis and Economics of Dairy Cows

**DOI:** 10.3390/ani10060937

**Published:** 2020-05-29

**Authors:** Nagwa I. El-Kasrawy, Ayman A. Swelum, Mervat A. Abdel-Latif, Abd El-Wahab A. Alsenosy, Noha A. Beder, Saad Alkahtani, Mohamed M. Abdel-Daim, Ayman H. Abd El-Aziz

**Affiliations:** 1 Animal Husbandry and Animal Wealth Development Department, Faculty of Veterinary Medicine, Damanhour University, Damanhour 22511, Egypt; nagwa.ibrahim@vetmed.dmu.edu.eg (N.I.E.-K.); ayman.sadaka@vetmed.dmu.edu.eg (A.H.A.E.-A.); 2 Department of Theriogenology, Faculty of Veterinary Medicine, Zagazig University, Sharkia 44519, Egypt; aymanswelum@zu.edu.eg; 3 Department of Animal production, College of Food and Agriculture Sciences, King Saud University, P.O. Box 2460, Riyadh 11451, Saudi Arabia; 4 Department of Nutrition and Veterinary Clinical Nutrition, Faculty of Veterinary Medicine, Damanhour University, Damanhour 22511, Egypt; 5 Biochemistry Department, Faculty of Veterinary Medicine, Damanhour University, Damanhour 22511, Egypt; dr_alsenosy_2010@yahoo.com; 6 Department of Animal Medicine, Faculty of Veterinary Medicine, Damanhur University, Damanhour 22511, Egypt; noha.abdallah@vetmed.dmu.edu.eg; 7 Department of Zoology, College of Science, King Saud University, P.O. Box 2455, Riyadh 11451, Saudi Arabia; salkahtani@ksu.edu.sa; 8 Department of Pharmacology, Faculty of Veterinary Medicine, Suez Canal University, Ismailia 41522, Egypt

**Keywords:** cattle, glycerol, propylene glycol, production, reproduction, subclinical ketosis

## Abstract

**Simple Summary:**

As the transition period is the most critical physiological stage of dairy cattle, propylene glycol as a gluconeogenic precursor was studied either individually or in combination with glycerol with different doses at different times. Their beneficial effects were recorded regarding productive and reproductive performances, protection against subclinical ketosis and economics.

**Abstract:**

A total of 108 Holstein cows were used to evaluate the effect of drenching propylene glycol (PG) either individually or in combination with glycerol (G) on body condition score (BCS), production, reproductive performance, protection against subclinical ketosis and economic benefit of dairy cows during the transition period. The six groups (n = 18/group) were; Control group, cows received no treatment; PG300, cows were drenched 300 mL PG for 7 days pre-expected day of calving and 21 days post-calving; PG400, cows were drenched 400 mL PG for 7 days pre-and 7 days post-calving; PG500, cows were drenched 500 mL PG for 10 days pre-and 10 days post-calving; GPG150, cows were drenched a mixture of 150 mL G and 150 mL PG for 21 days pre-and 21 days post-calving; GPG250, cows were drenched a mixture of 250 mL G and 250 mL PG for 21 days pre-and 21 days post-calving. BCS at 30 days in milk (DIM) was significantly higher in all treated groups in comparison to the control one and the best values were observed in GPG250, GPG150 and PG500 groups. Lactation length (LL) was significantly (*p* < 0.001) shorter in GPG250, GPG150 and PG500 groups than control group. There was a significant increment in 305 milk yield (*p* < 0.001) and average daily milk yield (*p* < 0.001) in GPG250 and PG500 groups than other groups except for PG300 with the lowest values for control and PG400. Cows in all treatment groups were observed in heat and conceived earlier as well as had shorter open days and calving interval durations (*p* < 0.001) and low number of services per conception (*p* = 0.004) compared to control group with better results for PG500 and GPG250 groups. BHB level and percentage of cows suffered from subclinical ketosis at 7 DIM was significantly lower in GPG250, GPG150 and PG500 groups than control group. Cows in treatment groups had a significantly higher glucose level (*p* = 0.006) compared to control group. Regarding to economics, the control group showed the highest feed costs, insemination costs and total costs per animal. Additionally, control and PG400 groups had the highest cost per kilogram of milk from total and feed costs compared to other cows. PG300, PG500 and GPG250 groups recorded a greater net return and income over feed cost (IOFC). In conclusion, the continuous drenching of propylene glycol either individually or in combination with glycerol regimens for long duration (PG300, PG500, GPG150, GPG250) during the transition period of dairy cows may reduce the incidence of subclinical ketosis and consequently improve milk production, reproduction and economics compared to drenching for short duration (PG400).

## 1. Introduction

Keeping profitability of production and reproduction is one of difficult assignments for dairy herds. The transition period is the most critical physiological stage for dairy cattle [1,2]. During this period, the feed intake decreases due to decreased rumen capacity during late gestation period due to fetus growth, and low post-calving appetite which combined with increased nutrients requirements for colostrum and milk synthesis. These issues force the transition cow to undergo negative energy balance (NEB) [3]. In a physiological adaptive process, the NEB stimulates lipomobilization in the form of non-esterified fatty acids (NEFA) and subsequently, beta-hydroxybutyrate (BHB) in the blood, which is considered the most abundant (78%) type of ketone bodies created by the liver, to overcome the deficient energy [4]. High level of ketone bodies in body fluids, is an indicative of excessive lipomobilization, and is often associated with inappetence and decreased milk yield. When dairy cattle failed to overcome this challenge, some metabolic and infectious disorders appeared which affect their productive and reproductive performance [5,6].

In a try to minimize the NEB, decrease lipomobilization, the incidence and severity of ketosis on dairy herds, evaluation of gluconeogenic precursors supplementation in transition cow diets has received a great attention [7,8,9]. Gluconeogenic precursors can stimulate gluconeogenesis, increase plasma glucose, and decrease lipolysis [10]. Propylene glycol (PG) and glycerol (G) are used as gluconeogenic precursors [9,11,12,13,14,15]. While, G infusion increases plasma glucose concentration faster than PG, PG maintained a higher plasma glucose concentration for a longer period of time after infusions than G [9,16]. However, large doses of PG (>500 g/day) can harm cattle that may be at least partially related to the production of sulfur-containing gases, emitted during fermentation of PG in the rumen [17,18]. PG can be either fermented to propionate in the rumen or absorbed and metabolized into glucose by the hepatic cells [19]. Even though, several experiments fed gluconeogenic precursor by top-dressing rations, few administered it as a drench [9]. Until recently, the price of G was expensive and not competitive with PG. However, its availability has increased from the expansion of the biodiesel industry, reducing its cost. Drenching of gluconeogenic precursors was better in stimulating plasma insulin and reducing plasma NEFA compared with its mixing in a total mixed ration (TMR) because of low appetite observed in these animals [20]. In addition, PG drenches increase glucose and insulin and decrease NEFA and BHB plasma concentrations more than G [21]. Most of previous researches used propylene glycol and/or glycerol as a drench in few doses especially after parturition [21]. While, other previous researches used propylene glycol as a powder top dressing; they used it for long duration extended to 28 days or more [8]. Few studies evaluated the effects of gluconeogenic precursor in the close- up period. Moreover, limited studies investigated the effect of different types and their combinations, doses and duration on efficiency of these anti-ketogenic additives during the transition period regarding to productive and reproductive parameters. Therefore, it is hypothesized that the type, amount and duration of drenching of gluconeogenic precursors can affect the reproductive and productive performances of dairy cattle. Therefore, the objective of the experiment under discussion was to study the efficacy of drenching PG or mixture of PG and G by different amount and for different durations on milk yield, reproductive performance, BCS, levels of BHB and glucose and economics of dairy cattle.

## 2. Materials and Methods

All experimental procedures involving animals were licensed according to the Local Experimental Animal Care Committee and approved by the ethics of the institutional committee of Animal Husbandry and Animal Wealth Development, Faculty of Veterinary medicine, Damanhour University, Egypt (DMU/VetMed-2019-/0145).

### 2.1. Animals, Management, Experimental Design and Treatment Regimen

This experiment was carried out on a commercial Holstein dairy farm located in Nubaria, Beheira Province, Egypt. A total of 108 transition pregnant cows, had only one previous lactation, were selected with average body weight (BW) and body condition score (BCS) of 524 ± 63 kg and 3.63 ± 0.2, respectively. The expected calving season of these cows was during winter (November-February) and all these cows were followed up for about one year (subsequent calving) to record their productive and reproductive data. Animals had free access to water, were fed ad libitum total mixed ration (TMR) twice according to recommendation of National research council (NRC). Ingredients and chemical analysis results of TMR fed to the animal throughout the experiment were illustrated in Table 1.

**Figure 1:**
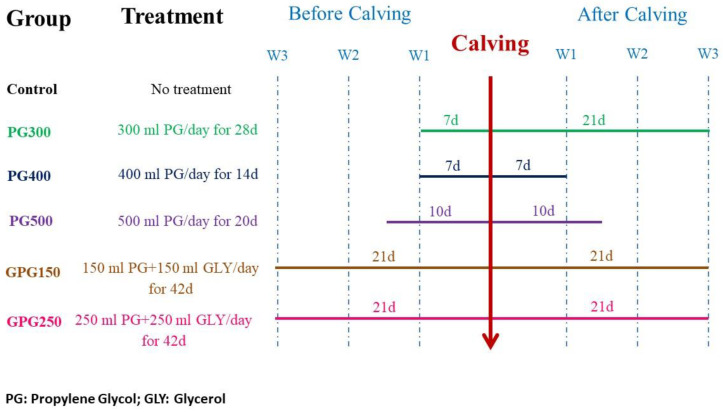
Experimental design and treatment regimen.

Animals were randomly and equally distributed into six groups (n = 18/group) according to regimen of treatment as follows: Control group, cows received no supplement; PG300, cows were daily drenched 300 mL propylene glycol (monohydrus, 99% purity, Qingdao Foture International Trade co., Ltd, Qingdao, China) for 7 days before expected day of calving (283 days gestation length) and 21 days after calving; PG400, cows were daily drenched 400 mL propylene glycol for 7 days pre-and 7 days post-calving; PG500, cows were daily drenched 500 mL propylene glycol for 10 days pre-and 10 days post-calving; GPG150, cows were daily drenched a mixture of 150 mL glycerol (99.7% purity, PT Willmar Nabati Indonesia Company, Medan, Indonesia) and 150 mL propylene glycol for 21 days pre-and 21 days post-calving; GPG250, cows were daily drenched a mixture of 250 mL glycerol and 250 mL propylene glycol for 21 days pre-and 21 days post-calving (Figure 1).

All cows were allowed a regular vaccination and deworming against the common epidemic and parasitic diseases. In addition, all cows were clinically observed daily by skilled veterinarians for any signs of illness and metabolic disorders especially ketosis.

### 2.2. Evaluation of Body Condition Score (BCS), Milk Productivity and Reproductive Traits

Body condition score (BCS) was evaluated by the same expert veterinarian for all experimental animals (3 times/observation) and was measured at day 0 (day of calving), day 30 and day 75 postpartum using a scale 1 (poor) to 5 (grossly fat) according to Roche et al. [22].

Animals were milked three times a day (2:00 a.m., 10:00 a.m., 6:00 p.m.). Milk yield per animal during the lactation season was automatically recorded through electronic milk meter fixed in each milking unit in the parlor and data transferred through network to the computer system. (ALPRO-Herd Management System-DeLaval Co, Sweden). Milk production of all animals was monitored from the current calving to the next one and the actual 305d milk yield (305-MY), the actual total milk yield (TMY), daily milk yield (DMY) and lactation length (LL) or days in milk (DIM) were recorded.

All cows were followed up during late gestation, parturition and puerperium period and all observations during these periods were recorded. Estrus was detected by pedometer records through high activity and cows thought to be in heat were inseminated intrauterine 12 h after onset of estrus using proved good quality semen (ABS, WWS, Alteagenitic) by an expert inseminator. Pregnancy diagnosis was done by rectal palpation 45 days post-insemination and then was confirmed rectally 90 days post-insemination. Pregnant cows followed up until its next calving. Days in milk first breeding (DIMFB), days open (DO), calving interval (CI) and number of service per conception (S/C) were recorded.

### 2.3. Blood Metabolites

Blood samples were collected (jugular vein) at day 7 and 14 postpartum and centrifuged (at 3000 rpm for 15 minutes) for serum collection and analysis of BHB and glucose levels, respectively. BHB concentrations in serum were analyzed using the Randox D-3 Hydroxybutyrate (Ranbut) assay and RX monza analyzer for quantitative in vitro determination of D-3-hydroxy butyrate (Ranbut; Randox Laboratories, Antrion, United Kingdom) [23]. An elevation of BHB concentration above 1.2 mmol/L (12.49 mg/dL) indicates subclinical ketosis in dairy cows [24]. Glucose was assayed by colorimetric method using a commercial Kit from Biodiagnostic Company (Dokki, Giza, Egypt) and UV-2100 UV/VIS Spectrophotometer (Shimadzu Corporation, Tokyo, Japan) [25]. Blood samples were classified according to its glucose level as hypo- (<40 mg/dL), normo- (40–60 mg/dL) or hyperglycemic (>60 mg/dL) [26].

### 2.4. Economic Assessments

Economic inputs (daily feeding cost, kilogram of milk price, price of semen straws, price of each supplement including period of treatment and other daily costs including drugs, disinfectants, veterinary supervision, labor cost and other miscellaneous costs have been spent on other facilities such as water and electricity etc,) were used to calculate the feeding cost, insemination cost, supplementation cost, total costs per animal, total cost per one kilogram of milk, feed cost per kilogram of milk, milk income, net return and milk income over feed cost. The calculated costs based on the prevailing prices during the period of the study and were calculated on the basis of Egyptian pound (EGP). The economic values were converted into US$ in exchange rate (1 US$ = 16.75 Egyptian pound (EGP). The cost of the supplement was set for entire administration period were (control group (0), 300 mL PG (18.05 US$/cow), 400 mL PG (12.04 UD$/cow), 500 mL PG (21.49 UD$/cow), 150 mL G + 150 mL PG (22.55 UD$/cow) and 250 mL G + 250 mL PG (37.59 UD$/cow). Feeding cost was calculated by multiplying average price of one kilogram dry matter intake (DM) by quantity of (DM) given to the animal. Semen costs were obtained by multiplying number of services by price of one straw of semen (8.35 US$). Total costs were calculated throughout the calving interval and they were calculated by summation of all production costs.

Milk income was calculated as the sum of milk produced per whole lactation multiplied by milk purchase price. Net return was calculated by subtraction of total costs from total sales of milk. Income over feed costs (IOFC) which used to measure income without fixed and labor costs, was calculated according to Jagannatha et al. [27] as difference between total milk income using mean milk price received during the trial (0.433 US$/kg) and total feeding costs.

### 2.5. Statistical Analyses

Milk yield, reproductive and economic parameters were analyzed by using a mixed model procedure (PROC MIXED), version 9.3 (SAS Institute Inc., Cary, NC, USA). The model for statistical analyses included the fixed effect of cow’s treatment regimens and the random effect of calving date. To analyze BCS and average daily milk yield data, repeated measurements was used using the previously mentioned model. The comparison of means was carried out with Duncan’s multiple range tests, after verifying normality using Kolmogorov-Smirnov test. Results were declared significant at *p* ≤ 0.05. The Chi-square test was used to evaluate the association between supplements and proportion of dichotomous variables (percentage of animals suffering from sub-clinical ketosis or hypoglycemia according to BHB or glucose levels, respectively).

## 3. Results

The cows were apparently healthy all over the period of trials. No signs of illness (including depression, ataxia, and excessive salivation, as well as abnormal, malodorous, and foul breath and feces) and/or metabolic disorders were observed in all cows.

### 3.1. Body Condition Score (BCS), Milk Productivity and Reproductive Traits

Effect of different drenching regimens by gluconeogenic precursors on BCS is shown in Table 2. The results revealed that, treatment and time had a significant (*p* < 0.001) effect on BCS and there was no significant effect of treatment by time interaction (*p* = 0.116). BCS was significantly higher in all groups at 0 DIM than at 30 DIM and 75 DIM. Regarding to effect of treatment, BCS didn’t differ among all groups at calving day. However, at 30 and 75 DIM, the BCS was significantly higher in all treated groups in comparison with control cows. The highest values were observed in cows treated with GPG250.

**Figure 2:**
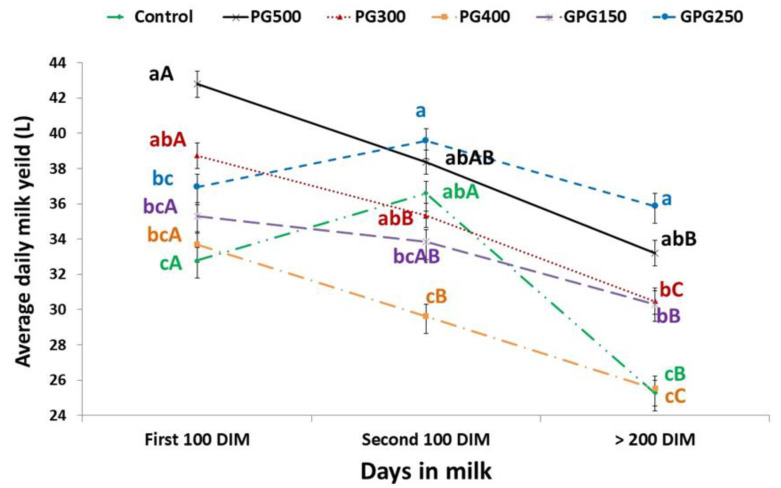
Effect of different drenching regimens by gluconeogenic precursors and time of assessment on average daily milk yield in first 100 DIM, in second 100 DIM and after 200 DIM. Means within duration with no common small superscript letters are significantly different (*p* ≤ 0.05) (treatment effect). Means within treatment with no common capital superscript letters are significantly different (*p* ≤ 0.05) (time effect). The probability values of treatment effect, time effect and their interaction were <0.001, <0.001 and <0.001, respectively.

The effects of different drenching regimens by gluconeogenic precursors on milk production parameters, including lactation length (LL), total milk yield (TMY), 305-d milk yield (305-MY) and daily milk yield (DMY) were illustrated in Table 3. Our results revealed that TMY were not affected by the levels of the supplement. Lactation length was significantly shorter in GPG250, GPG150 and PG500 groups than that of the control group. The 305-MY and average daily milk yield were significantly higher in GPG250 and PG500 groups than in other groups except PG300 with the lowest value for control and PG400. Average daily milk yield during first 100 days and second 100 days and after 200 days was shown in Figure 2, where treatment, time and their interaction had significant (*p* < 0.001) effect on average daily milk yield in first 100 DIM, in second 100 DIM and after 200 DIM. PG500 group showed the highest daily production in first 100 days in milk, while GPG250 group was the highest in second 100 days in milk and later compared to other groups. Regarding to time effect, the lowest average daily milk yield was observed after 200 DIM in all groups. 

All cows had normal parturition, live calf and without retention of the placenta or puerperium infection. Effect of different drenching regimens by gluconeogenic precursors on reproductive performance is presented in Table 4. The results revealed that cows in all treatment groups showed an improvement in DIMFB, DO, CI and SC compared to cows in control group. The cows in GPG250 group had the shortest days open and calving interval durations than other groups. The number of service per conception was significantly lower in cows of PG300, PG500 and GPG250 groups than control group.

### 3.2. Blood Metabolites

Effect of different drenching regimens by gluconeogenic precursors on serum levels of BHB and glucose, is clarified in Table 5. Results revealed that BHB at 7 DIM was significantly lower in GPG250, GPG150 and PG500 groups than control one. The lowest value was observed in GPG250 group. Furthermore, all treated groups showed a significant increase in glucose level at 14 DIM than control one. Percentage of cows suffered from SCK and hypoglycemia was significantly decreased in all treatment groups in comparison to control group.

### 3.3. Economic Parameters

The economic results of different evaluated groups on the production costs and returns were existed in Table 6. Results of the current study revealed that the highest feed cost, insemination cost and total cost per animal were for control group. Also, the highest total cost and feed cost per kilogram milk were seen for control and PG400 groups. Regarding the return parameters, no significant difference existed in milk return per animal. Besides, the greatest net return and IOFC were found in PG300, PG500 and GPG250 groups.

## 4. Discussion

The nutrition and management of dairy cows during the transition period is very important. Therefore, many researches focused on using glycerol and/or propylene glycol as drench or feed additives to reduce NEB and ketosis incidence [5,17]. Glycerol and propylene glycol are gluconeogenic products, energy-rich components and used as anti-ketogenic in transition period. Dosage of PG is restricted (≤500 g/day) for its potential toxic effects [17]. Glycerol is safe to be administered as drench in large amount [16]. However, the high cost limited its use in dairy farms [28,29]. The goals of treatment by G and/or PG are to stimulate gluconeogenesis, increase plasma glucose, decrease lipolysis and thereby reduce incidence of ketosis [10].

In our result, PG500, GPG150 and GPG250 groups showed significant decreases in serum concentration of BHB at 7 DIM with the lowest value in GPG250, and significant increases in blood level of glucose at 14 DIM compared to control group. The percentage of cows suffered from SCK and hypoglycemia was significantly decreased in all treatment groups in comparison to control group. Interestingly, no cow suffered from subclinical ketosis at 7 DIM or hypoglycemia at 14 DIM was observed in GPG150 and GPG250 groups. This means that PG alone or its mixture with G has been effective in reducing lipolysis and BHB level and increasing glucose level and then, reducing the incidence of ketosis. These blood biochemical changes were reflected in cows’ reproductive and productive performance.

Similar results were reported by many previous researches. Chung et al. [8] concluded that dry PG supplement as a top dress decreased BHB concentrations and subsequently reduced the incidence of subclinical ketosis. Kristensen et al. [30] suggested that infusion of 650 mL (mL/kg wt) PG to the rumen of lactating cows increases plasma glucose concentration as it increases gluconeogenic precursors and induces insulin resistance. On the other hand, G increased glucose and decreased NEFA and BHB plasma concentrations after daily (400 mL pure G/day) oral administration during the first 14 DIM compared to the control group [16]. In another study, G decreased plasma NEFA after daily (500 g G (82.6% purity)/day) oral administration during the first 21 DIM, but did not affect other metabolites compared to the control group [31]. Additionally, Piantoni and Allen [9] concluded that dosing 300 mL of PG to the cranial reticulo-rumen is more effective for ketosis treatment than dosing the double quantity of G due to the extensive metabolism of G by ruminal microbes to metabolites that are not glucogenic and added that adding G to the recommended dose of PG is doubtful to provide additional benefit. Intravenous administration of glycerol and PG effectively reduced hyperketonemia and lipolysis [32].

Our results revealed that, cows in all supplemented groups were observed in heat and conceived earlier as well as had shorter days open and calving interval durations than the cows in control group. This may be attributed to the cows in control group were suffered from excessive NEB which can be observed by monitoring BCS following calving. The current results showed that at 30 DIM the BCS was significantly higher in all treatment groups than control group. It is known that excessive BCS loss during the first 30 DIM is associated with delayed ovulation and poor fertility. This can also explain why the best DIMFB, DO, CI and S/C were observed in GPG250 group followed by PG500 group which had the best BCS at 30 DIM. The postpartum body condition score in lactating animals is related to the interval to conception [33]. Generally, the best BCS (2.5–4) near AI has a positive effect on the productive efficiency of lactating cows. Additionally, poor BCS during the early lactation has a negative effect on pregnancy at first calf heifer [34]. Lower BHB concentrations (plasma BHB level ˃1.56 mg/dL) were better for rapid recovery of ovarian activity in primiparous and multiparous cows [35,36].

Similarly, earlier first ovulation, higher conception rate at first insemination and longer luteal phase were reported in cows drenched 500 mL PG for 7 to 42 DIM compared with control cows [37]. On the contrary, no changes were found in reproductive performance of cows drenched daily 500 mL PG 10 d pre to 25 d post-partum [5]. Reproductive organs and processes including oocyte development require glucose [38]. Hypoglycemia in the early lactation stage indicates an energy priority to lactation over reproduction [39]. Short lactation length in GPG250 and PG500 groups considers normal result for early conception. In spite of short lactation length in GPG250 and PG500 groups, the highest 305-MY value was observed in these two groups due to the highest average DMY. The average DMY during first 100 DIM and second 100 DIM was observed in GPG250 and PG500 groups which means that the cows in these groups reach the peak of milk yield earlier than other groups with high persistency. Similar results in TMY because groups that had low DMY had long lactation length. Increasing effect of different gluconeogenic precursors on milk yield was previously reported [8,40] but PG had no effect on either milk yield or its constituents [17,41]. These results may due to cows were at positive energy balance. The cause of difference between the results obtained after using gluconeogenic products may be attributed to many factors including: type, dose, duration, and methods of application. Drenching of gluconeogenic products especially PG, remains the most effective method without effect on feed intake compared to its incorporation in TMR.

High proportion of gluconeogenic precursors can escape microbial fermentation and degradation of the rumen and absorbed intact in the small intestine [42]. The rest of G is metabolized by rumen microbes mainly to propionate, butyrate, and acetate [8,43] whereas the rest of PG is mainly metabolized into propionate, propanol, and propanal [30]. By increasing the dose of gluconeogenic precursors, the acetate to propionate ratio is decreased indicating ruminal conversion of gluconeogenic precursors to propionate [20]. Finally, propionate is the primary glucogenic substrate in the dairy cow [6]. In the liver, PG is metabolized into lactate and subsequently to glucose [30] which can support the fetal unit of placenta during gestation [4]. Constantly, the lactate response to G was minor compared to its major response to PG and then the faster drop in BHB level was observed in response to PG as compared to G. However, the more powerful stimulation of insulin release was observed in G when compared by PG and contributed significantly to the reduction of BHB level [32]. Glycerol can alleviate the hyperketonemia by substantial hyperglycemia and consequently, increases the capacity of oxidation of acetyl-CoA via the tricarboxylic acid (TCA) TCA cycle which depletes precursors for ketone bodies synthesis [17]. As a result, PG and G have different pathways to be metabolized into glucose. Therefore, their mixture in GPG250 is beneficial for hepatic synthesis of glucose [32,40].

The improvement of production and reproduction in the treated groups was reflected on the economic benefit. Increasing days open, lactation length and calving interval in control group caused increasing in the cost specially feed cost. Increasing milk yield caused increasing in return. Economic losses as a result of ketosis were estimated to total approximately $145/case, which included decreased milk production, increased days open, treatment of clinical cases and increased culls [44]. It is worth mentioning that, the beneficial effect in GPG250 is mainly due to PG supplementation. These findings was supported by the results of Pechova et al [45] who found that, there was no beneficial effect to glycerol above 500–1000 mL. Also, they reported that 500 mL of glycerol was effective as 300 mL propylene glycol. The best mixture of PG and G need further research and also the maximum drenching dose of PG and time interaction is still unknown.

## 5. Conclusions

Drenching PG300, PG500 and GPG250 can improve milk production. All treated groups recorded better reproductive performance and higher glucose level than control. The best values of BCS at 30 DIM and the lowest BHB level were for PG500 and GPG mixture groups. Regarding to economics, control cows showed the highest feed costs, insemination costs and total costs. Cost per kilogram of milk from total and feed costs was higher in control and PG400 groups compared to other treated cows. The highest net return and income over feed cost (IOFC) was observed in PG300, PG500 and GPG250 groups. Therefore, we recommend usage of PG300, PG500 and GPG mixture during transition period of dairy cows for improving of production, reproduction, and economics and reducing incidence of subclinical ketosis.

## Figures and Tables

**Table 1:** Ingredients and chemical analysis of total mixed ration.

Ingredient as Fed (Kg/Head/Day)	Close Up	Milking
Alfalfa hay	2.25	4.00
Berseem fresh	5.00	18.00
Corn Silage	16.00	15.25
Wheat straw	-	0.75
Total Forage	23.25	38.00
Ground yellow corn	3.50	7.00
Soybean 48%	0.20	3.00
Corn gluten feed	1.50	3.50
Rice Polish	0.70	2.00
Wheat bran	0.25	1.90
Molasses, cane	-	0.50
Magnapac	-	0.45
Limestone	-	0.090
Sodium bicarbonate	-	0.22
Mentrax (Chelated)	0.01	0.001
NEW T-NIL^®^ Dry ^1^ (Antimycotoxin)	0.03	0.04
Premix ^2^	0.03	0.1
Nutrients (%) according to feed analysis		
Crude Protein	15.2	18.2
NDF ^3^	34.43	33.71
ADF ^4^	23.01	17.63
Cellulose	18.6	15.2
Hemicellulose	11.42	16.08
Lignin	4.41	2.43
NDICP ^5^	2.84	2.26
ADICP ^6^	1.75	0.71
Crude Fat	2.29	3.88
Ash	10.96	9.03
NFC ^7^	34.38	34.83
NEl(Mcal/kg) ^8^	1.5	1.64

^1^ Ascorbic acid 0.05%, Citric acid 0.75%, Calcium propionate 10.5%, Copper sulphate 5%, Inactivated yeast (*Saccharomyces Cerevisiae*) 2%, Sapiolite 41.7%, Bentonite 40%. ^2^ (Vit. A 10,000,000 IU, Vit D3 2,500,000 IU, Vit. E 35,000 mg, Biotin 1000 mg, Zinc 100,000 mg, Mn 80,000 mg, Cu 30,000 mg, I 800 mg, Co 400 mg, Se 300 mg, CaCO_3_ to 3 kg). ^3^ Neutral Detergent Fiber; ^4^ Acid Detergent Fiber; ^5^ Neutral Detergent Insoluble Crude Protein; ^6^ Acid Detergent Insoluble Crude Protein; ^7^ Non Fibrous Carbohydrates; ^8^ Net Energy for Lactation.

**Table 2:** Effect of different treatment regimens by gluconeogenic precursors and the time of assessment on body condition score (BCS).

Item	Control	Gluconeogenic Precursors Supplementation					SEM	*p*-Value *
		PG300	PG400	PG500	GPG150	GPG250		
BCS 0 DIM	3.56 ^A^	3.53 ^A^	3.63 ^A^	3.59 ^A^	3.69 ^A^	3.61 ^A^	0.03	0.116
BCS 30 DIM	2.45 ^d,B^	2.62 ^b,c,B^	2.55 ^c,B^	2.77 ^a,b,B^	2.78 ^a,b,B^	2.81 ^a,B^	0.02	0.008
BCS 75 DIM	2.42 ^b,B^	2.52 ^a,b,B^	2.50 ^a,b,B^	2.59 ^a,b,B^	2.56 ^a,b,B^	2.65 ^a,B^	0.02	0.011
SEM	0.07	0.08	0.06	0.09	0.10	0.06		
*p*-Value **	<0.001	<0.001	<0.001	<0.001	<0.001	<0.001		

Means within each row carrying different small superscripts are significantly different (*p* ≤ 0.05). Means within each column carrying different capital superscripts are significantly different (*p* ≤ 0.05). SEM: standard error of the mean. DIM: days in milk. * Effect of treatment. ** Effect of time. The probability values of treatment effect, time effect and their interaction were <0.001, <0.001 and 0.116, respectively.

**Table 3:** Effect of different drenching regimens by gluconeogenic precursors on productive performance.

Item	Control	Gluconeogenic Precursors Supplementation					SEM	*p*-Value *
		PG300	PG400	PG500	GPG150	GPG250		
LL	377.83 ^a^	342.31 ^a,b^	346.39 ^a,b^	308.10 ^b,c^	335.67 ^b,c^	299.50 ^c^	5.69	<0.001
TMY	11433.14	11744.65	10069.55	11701.63	11030.11	11225.26	213.18	0.216
305 MY	9229.30 ^b,c^	10464.55 ^a,b^	8866.35 ^c^	11583.90 ^a^	10022.30 ^b,c^	11431.14 ^a^	210.78	<0.001
DMY	30.26 ^b,c^	34.31 ^a,b^	29.07 ^c^	37.98 ^a^	32.86 ^b,c^	37.48 ^a^	0.69	<0.001

Means with different superscripts in the same row are significantly different (*p* ≤ 0.05). SEM: standard error of the mean LL: lactation length, TMY: actual total milk yield, DMY: average daily milk yield during whole lactation. 305 MY: milk yield in 305 days. * Effect of treatment.

**Table 4:** Effect of different drenching regimens by gluconeogenic precursors on reproductive performance.

Item	Control	Gluconeogenic Precursors Supplementation					SEM	*p*-Value *
		PG300	PG400	PG500	GPG150	GPG250		
DIMFB	92.89 ^a^	65.85 ^b^	69.17 ^b^	62.70 ^b^	65.44 ^b^	54.11 ^b^	2.87	<0.001
DO	185.89 ^a^	127.23 ^b,c^	141.89 ^b^	97.50 ^b,c^	115.67 ^b,c^	81.78 ^c^	7.03	<0.001
CI	467.89 ^a^	409.23 ^b,c^	423.89 ^b^	379.50 ^b,c^	397.67 ^b,c^	363.78 ^c^	7.03	<0.001
S/C	3.83 ^a^	2.62 ^b^	2.78 ^a,b^	1.90 ^b^	2.67 ^a,b^	2.00 ^b^	0.16	0.004

Means with different superscripts in the same row are significantly different (*p* ≤ 0.05). SEM: standard error of the mean, DIMFB: days in milk at first breeding, DO: days open. CI: calving interval, S/C: number of services per conception. * Effect of treatment.

**Table 5:** Effect of different treatment regimens on serum levels of BHB and glucose (mg/dL) as well as the percentage of cows suffered from subclinical ketosis (SCK) (>12.49 mg/dL) or hypoglycemia (<40 mg/dL).

Item	Control	Gluconeogenic Precursors Supplementation					SEM	*p*-Value	Chi Square Value:	
		PG300	PG400	PG500	GPG150	GPG250				
At 7 DIM	% of cows suffered from SCK (number)	61.1 (11)	27.7 (5)	33.3 (6)	16.6 (3)	0 (0)	0 (0)		0.012	14.56
	BHB	13.71 ^a^	12.16 ^a,b^	12.02 ^a,b^	10.26 ^b,c^	9.52 ^c^	7.42 ^d^	0.35	<0.001	
At 14 DIM	% of cows suffered from hypoglycemia (number)	18.2 (4)	0 (0)	0 (0)	0 (0)	0 (0)	0 (0)		0.005	16.97
	Glucose	43.85 ^b^	51.77 ^a^	49.16 ^a,b^	53.33 ^a^	54.50 ^a^	51.75 ^a^	0.96	0.006	

Means with different superscripts in the same row are significantly different (*p* ≤ 0.05). SEM: standard error of the mean.

**Table 6:** Effect of different drenching regimens by gluconeogenic precursors on cost and return parameters.

Item.	Control	Gluconeogenic Precursors Supplementation					SEM	*p*-Value
		PG300	PG400	PG500	GPG150	GPG250		
Feed Cost/Animal (US$)	2779.95 ^a^	2526.20 ^a,b^	2520.86 ^a,b^	2344.21 ^b^	2486.60 ^b^	2271.12 ^b^	38.45	0.003
Insemination Cost/Animal (US$)	31.98 ^a^	21.87 ^b^	23.21 ^a,b^	15.87 ^b^	22.29 ^a,b^	16.70 ^b^	1.39	0.007
Total Cost/Animal (US$)	3510.33 ^a^	3176.91 ^b^	3188.78 ^b^	2948.00 ^b^	3124.97 ^b^	2868.37 ^b^	49.10	<0.001
Cost per kg Milk (US$)	0.31 ^a^	0.27 ^a,b^	0.32 ^a^	0.25 ^b^	0.28 ^a,b^	0.26 ^b^	0.01	<0.001
Feed cost per kg Milk (US$)	0.24 ^a^	0.22 ^a,b^	0.25 ^a^	0.20 ^b^	0.23 ^ab^	0.20 ^b^	0.04	<0.001
Milk Return (US$)	4950.54	5085.43	4360.12	5066.8	4776.04	4860.53	92.30	0.213
Net Return (US$)	1440.21 ^b,c^	1908.52 ^a,b^	1171.34 ^c^	2118.80 ^a^	1651.07 ^b,c^	1992.16 ^a^	83.00	<0.001
IOFC (US$)	2170.59 ^a,b^	2559.23 ^a^	1839.26 ^b^	2722.59 ^a^	2289.44 ^a,b^	2589.41 ^a^	81.44	0.029

Means within each row for each division with no common superscript letters are significantly different (*p* ≤ 0.05). SEM: standard error of the mean. IOFC: milk income over feed cost.
